# Subclinical and Clinical Outcomes in Patients Coinfected With HIV and Chronic Hepatitis B Virus From Clinical Outpatient Centers in France: Protocol for an Ambispective, Longitudinal Cohort Study

**DOI:** 10.2196/24731

**Published:** 2021-04-06

**Authors:** Anders Boyd, Lorenza N C Dezanet, Raisha Kassime, Patrick Miailhes, Caroline Lascoux-Combe, Julie Chas, Pierre-Marie Girard, Joël Gozlan, Fabien Zoulim, Constance Delaugerre, Hayette Rougier, Karine Lacombe

**Affiliations:** 1 Sorbonne Université, INSERM Institut Pierre Louis d'Épidémiologie et de Santé Publique Paris France; 2 Service de Maladies Infectieuses et Tropicales, Hôpital de la Croix-Rousse, Hospices Civils de Lyon Lyon France; 3 Service de Maladies Infectieuses, Hôpital Saint-Louis, APHP Paris France; 4 Service de Maladies Infectieuses, Hôpital Tenon, APHP Paris France; 5 Service de Maladies Infectieuses, Hôpital Saint-Antoine, APHP Paris France; 6 Centre de Recherche Saint-Antoine Paris France; 7 Centre de Recherche sur le Cancer de Lyon, Unité 1052, INSERM, UMR 5286, CNRS Lyon France; 8 Laboratoire de Virologie, Hôpital Saint-Louis, APHP; Université de Paris, INSERM U944, Institut de Recherche Saint-Louis Paris France; 9 Institut de Médecine et d’Epidémiologie Appliquée (IMEA) Paris France

**Keywords:** liver cirrhosis, hepatitis B, viral load, longitudinal studies, immunosuppression

## Abstract

**Background:**

Previous large-scale studies have examined the effect of chronic hepatitis B virus (HBV) infection on overall and cause-specific mortality in individuals with HIV. However, few studies have collected data on the subclinical indicators of HBV that lead to these severe outcomes in the coinfected population.

**Objective:**

In this study, we aim to describe the procedures of a cohort study extension aimed at assessing HBV-DNA replication, serological markers of HBV (hepatitis B *e* antigen [HBeAg] and hepatitis B surface antigen), and liver fibrosis and how these subclinical outcomes relate to mortality in predominately tenofovir-treated, coinfected patients with HIV-HBV. We assessed the characteristics at cohort inclusion of those who participated in the cohort extension, as well as those who did not participate due to being lost to follow-up or death.

**Methods:**

Patients with HIV and chronic HBV who completed follow-up in a prospective cohort study conducted in 4 outpatient centers (Paris and Lyon, France; 2002-2011) were invited to participate in a cross-sectional visit from November 2016 to March 2018, during which a comprehensive evaluation of HIV- and HBV-related disease was undertaken. Virological and clinical data since the previous study visit were retrospectively collected.

**Results:**

Of the 308 individuals enrolled in the cohort, 147 (47.7%) participated in the cross-sectional study. At this visit, most participants were HBeAg negative (111/134, 82.8% with available data), had undetectable HBV DNA (124/132, 93.9% with available data), and were undergoing antiretroviral therapy containing tenofovir disoproxil fumarate or tenofovir alafenamide (114/147, 77.6%). There were no significant differences in characteristics at cohort inclusion between those who did and did not complete the cross-sectional visit, except for a lower proportion with an AIDS-defining illness (30/147, 20.5% vs 49/161, 30.4%*,* respectively; *P*=.04). Of the 161 nonparticipating individuals, 42 (26.1%) died, 41 (25.4%) were lost to follow-up and known to be alive, and 78 (48.4%) were lost to follow-up with unknown vital status. Most differences in characteristics at cohort inclusion were observed between deceased individuals and those participating in the cross-sectional visit or those lost to follow-up. With this extension, the median follow-up time of the overall cohort is presently 9.2 years (IQR 3.4-14.6).

**Conclusions:**

Extended follow-up of the French HIV-HBV cohort will provide important long-term data on the subclinical trajectory of HBV disease in the coinfected population. The biases due to the relatively high rate of those lost to follow-up need to be assessed in future studies of this cohort.

**International Registered Report Identifier (IRRID):**

DERR1-10.2196/24731

## Introduction

### Background

With the advent of potent antiretroviral therapy (ART), the incidence of death related to AIDS has substantially declined among individuals with HIV, leading to new causes of mortality [1]. Liver-related death has been topmost among these recent causes, due in part to chronic hepatitis B virus (HBV) coinfection, and it has been a major concern over the past decade [2].

Because high levels of plasma HBV replication are associated with liver cirrhosis, hepatocellular carcinoma, liver-related death, and overall mortality [3], the suppression of circulating virus is the primary goal of therapy. The potent anti-HBV agent tenofovir (TFV), which is able to extensively suppress HBV replication and be easily incorporated in most ART regimens, has been available since 2002 [4]. Nevertheless, there are conflicting data regarding whether the risk of severe liver-related morbidity (ie, hepatic decompensation, portal hypertension, end-stage liver disease, and hepatocellular carcinoma) and mortality are indeed reduced in coinfected versus mono-infected patients despite the use of this agent [5-10].

To characterize the risk of severe liver-related morbidity and mortality over time in the coinfected population, an adequate evaluation of HBV-DNA viral replication, HBV serological battery, and liver fibrosis is needed. These parameters are important subclinical indicators of chronic HBV disease. Unfortunately, many hospital-based cohorts, including individuals with HIV, do not have regularly collected HBV virological and serological data, either due to structural or cost issues [11-13]. Furthermore, most HIV-HBV cohort studies presently include either retrospective data or small patient sizes, wherein less potent anti-HBV agents, such as lamivudine, were administered [14-16]. Therefore, there is a need for cohort studies to collect more exhaustive data on subclinical indicators of HBV in the HIV-HBV coinfected population, especially during TFV-containing ART.

It was in this context that our research group initiated the French HIV-HBV cohort, which followed coinfected patients between 2002 and 2011 [17]. We recently invited participants of the cohort to return for a study visit in 2017-2018 with the purpose of (1) performing a cross-sectional, comprehensive evaluation of viral hepatitis and liver fibrosis and (2) retrieving biological and clinical data since their previous study visit in the cohort. The primary research aim of the cohort is to assess HBV-DNA replication, serological markers of HBV (hepatitis B *e* antigen [HBeAg] and hepatitis B surface antigen [HBsAg]), and liver fibrosis and assess how all 3 of these outcomes relate to mortality in predominately TFV-treated, coinfected patients with HIV-HBV.

### Objectives

In this paper, we present a protocol of this cohort. First, we aimed to expand on the procedures of the extension of this cohort study. The individual characteristics of the overall cohort were described and compared between those who did and did not participate in the cross-sectional visit mentioned earlier. Second, to provide more accurate information on mortality, we described the procedures by which we obtained the vital status of individuals who were lost to follow-up and assessed the number of cohort participants lost to follow-up. The characteristics of individuals who continued follow-up, who were lost to follow-up, and who were deceased were then compared. Finally, we discussed the importance of these data and how they will be used in future research.

## Methods

### Study Design and Visits

The French HIV-HBV cohort is a closed, prospective, longitudinal cohort study that included patients initially from 7 academic hospital centers located in Paris and Lyon, France, and is aimed at evaluating the determinants of liver-related morbidity and mortality in individuals coinfected with HIV-HBV. The inclusion criteria were HIV-positive serology confirmed by western blot, HBsAg-positive serology for at least 6 months, a Karnofsky score ≥70, aged 18 years or older, and provided signed written informed consent. Exclusion criteria were acute HBV infection (ie, HBsAg-positive for less than 6 months); any severe physical, clinical, or mental condition preventing participation (as determined by a Karnofsky score ≤70 or from recommendations by the treating physician); not fulfilling all inclusion criteria; or refusal to participate. Patient recruitment and follow-up of the cohort occurred in 3 phases, with the first 2 phases previously described in detail [17]. Table 1 shows the general characteristics of each study phase.

**Table 1:** General information at each phase of data collection.

Study component		Phase 1 (N=308)	Phase 2 (n=185)	Phase 3 (n=148)
Design		Prospective	Prospective	Cross-sectional and retrospective
Study visits		Routine clinical evaluation every 3 months; virological data collected every 6 months; fibrosis or serological data collected every 12 months	Routine clinical evaluation and data collection every 6 and 12 months for participants with F3/F4 and F0/F1/F2 fibrosis, respectively^a^	Cross-sectional visit including comprehensive clinical evaluation on viral hepatitis and liver fibrosis; retrospective data collected since last visit in phase 1 or 2
Inclusion period		2002-2003	2007-2008	2016-2018
Follow-up period		2002-2006	2007-2011	N/A^b^
Number of patients completing follow-up, n (%)		275 (89.3)	163 (88.1)	147 (99.3)
**Number of deaths**				
	Total	3	8	31
	Observed	3	6^c^	26^c^
	Updated^d^	0	2	5
Follow-up time (years), median (IQR)		3.0 (3.0-3.2)	7.3 (3.1-8.0)^e^	9.2 (3.4-14.6)^e^

^a^In accordance with the European AIDS Clinical Society guidelines for the clinical management and treatment of chronic hepatitis B and C coinfection in adults with HIV [18].

^b^N/A: not applicable.

^c^Includes deaths during time gaps in between phases.

^d^Using linked data from a national death certificate registry (CépiDC).

^e^Cumulated over previous phases—this statistic applies to all 308 patients regardless of the phase in which they discontinued participation.

In the first phase, 308 patients were recruited between May 2002 and May 2003 and prospectively followed every 3 months until the month-36 visit (2005-2006). In the second phase, 185 patients completing follow-up approximately 12 to 24 months after the first phase were recruited between March 2007 and March 2008. Patients with METAVIR F3/F4 fibrosis continued study visits every 6 months and patients with METAVIR F0/F1/F2 continued study visits every 12 months until the month-36 visit (2010-2011).

In the third phase, individuals completing follow-up in either the first or second phase were invited from November 2016 to March 2018 to participate in a cross-sectional study visit, which included a comprehensive evaluation of viral hepatitis and liver fibrosis. Approximately 4 mL of additional serum and plasma samples were collected and stored at −20 °C in a centralized location (Tumerothèque, Hôpital Saint-Antoine, Paris, France). Data were also retrospectively collected from the most recent study visit of the first or second phase until the cross-sectional visit or, in the case of death, until the most recent visit before death. The retrospective visits were selected from a 3-month period before or after the date of available HBV-DNA viral load (VL) measurement or, if missing, from a 3-month period before or after the date of the yearly visit.

All patients provided written informed consent at the beginning of each study phase. Retrospective data from deceased individuals were collected only if the patient had signed consent for the use of their clinical data during noninterventional research, consistent with the French Public Health Law. Protocols for each study phase were approved by the hospital ethics committee (first phase: Hôpital Pitié-Salpêtrière; second phase: Hôpital Saint-Antoine; third phase: Hôpital Hôtel-Dieu) in Paris, France, in accordance with the Helsinki Declaration.

### Primary Outcome Measures

A total of 4 primary outcomes were studied using data from this cohort extension.

#### Plasma HBV-DNA VL

This outcome was determined at inclusion and every 6 months in the first phase, every 6 to 12 months in the second phase, and in all retrospective and cross-sectional visits of the third phase. Commercially available polymerase chain reaction (PCR)–based assays were used to quantify HBV-DNA VL (COBAS AmpliPrep/COBAS TaqMan, detection limit: 12 international units [IU]/mL or COBAS Amplicor HBV Monitor, detection limit: 60 IU/mL; Roche Diagnostics).

We intend to study HBV DNA as continuous VLs, as undetectable HBV-DNA VLs, and as cumulative time-averaged HBV-DNA VLs [17].

#### HBeAg and HBsAg Seroclearance

This outcome was obtained from a complete HBV serological battery, which was performed at inclusion and every 12 months in the first phase, every 6 to 12 months in the second phase, and all retrospective and cross-sectional visits of the third phase. This consisted of qualitative HBsAg, HBeAg, anti–hepatitis B surface antibody (HBsAb), and anti-HBe antibody (HBeAb) detected using a commercial enzyme immunoassay (DiaSorin; Monolisa HBsAg, Bio-Rad; or Architect, Abbott Diagnostics).

We intend to study HBeAg seroclearance, defined as the transition from HBeAg-positive to HBeAg-negative status during follow-up in patients with HBeAg, and HBsAg seroclearance, defined as the transition from HBsAg-positive to HBsAg-negative status during follow-up in all included patients. We also aim to study HBeAg-seroconversion (ie, HBeAg seroclearance while acquiring anti-hepatitis B *e* antibody) in individuals with HBeAg and HBsAg-seroconversion (ie, HBsAg seroclearance while acquiring anti-HBsAb).

#### Liver Fibrosis

This outcome will be determined from several sources. First, several noninvasive biochemical scores predicting liver fibrosis levels were collected at inclusion and every 12 months in the first phase, every 6 to 12 months in the second phase, at the discretion of the treating physician in the third phase retrospective visits, and at the third phase cross-sectional visit. These scores included the FibroTest (Bio-Predictive), Fibrometre (Liver-Gastroenterology Department, CHU Angers), and Hepascore, all of which have been validated for use in the HIV-HBV coinfected population [19]. Second, measures of transient elastography (TE) were obtained at the physicians’ discretion in the first phase, every 6 to 12 months in the second phase, at the physicians’ discretion in the third phase retrospective visits, and at the third phase cross-sectional visit. TE was obtained using FibroScan (EchoSens) with either M or XL probes. Only TE measures fulfilling reliability criteria, as established by the manufacturer, were retained (ie, ≥10 valid measurements, IQR <30% of median stiffness, or ≥60% success rate) [20]. Shear wave elastography was also performed for some participants using the 2D real-time shear wave (Aixplorer, SuperSonic Imaging SA) with a 3.5 MHz convex ultrasound (SCX-6-1) and 7.5 MHz linear ultrasound (SL-10-2) probes.

We intend to study liver fibrosis as a continuous measure (in which case the analysis will only focus on one noninvasive measure) or at validated thresholds of METAVIR F3-F4 liver fibrosis [19] (in which case noninvasive measures can be combined, eg, any score indicating F3-F4 fibrosis).

#### All-Cause Mortality

Deaths observed during follow-up in the first and second phases, along with the underlying cause of death, were reported by the treating physician. At the third phase cross-sectional visit, treating physicians were asked to ascertain the vital status (ie, alive, deceased, or unknown) of patients completing follow-up in the first or second phases. If death occurred, they were requested to provide further information on the date and underlying cause of death.

To obtain vital status for patients with unknown status (ie, lost to follow-up), a trusted third party (Inserm U1018) was requested to link data from the French HIV-HBV cohort to a national identification registry (*Répertoire national d’identification des personnes physiques*, fichiers n° 779 and 801). For deceased individuals, the cause of death was then obtained by a separate trusted third party (CépiDC), linking data from the French HIV-HBV cohort to a national registry of death certificates (*certificat médical*) and death notifications (*bulletin d’Etat civil de décès*). Both registries are managed by the *Institut National de la Statistique et des Etudes Economiques*.

We intend to use all-cause mortality as an outcome of the analyses. Given the expected number of deaths, we will likely be unable to study the specific causes of death; however, these will be described in detail.

### Covariables

#### Laboratory Measures

Other laboratory measures, as listed below, were performed on blood samples taken during the study visits.

Antibodies to hepatitis C or D virus were detected using a commercial enzyme immunoassay at inclusion and every 12 months in the first and second phases, at the physician’s discretion during the third phase retrospective visits, and at the third phase cross-sectional visit. Serum hepatitis C virus (HCV) RNA and/or hepatitis D virus (HDV) RNA were quantified if the corresponding antibody test was positive (for the first and second phase and at the third phase cross-sectional visit) at the discretion of the treating physician (for the third phase retrospective visits). HCV RNA VLs were determined using either a PCR-based assay (COBAS Amplicor HCV Monitor v2.0, detection limit: 60 IU/mL; COBAS AmpliPrep/COBAS TaqMan HCV, detection limit: 10 IU/mL, Roche Diagnostic Systems; Abbott RealTi*m*e HCV, detection limit: 12 IU/mL, Abbott Molecular Inc) or branched-DNA technique (VERSANT HCV 3.0, detection limit: 615 IU/mL, Bayer Diagnostics). HDV-RNA VLs were quantified using a real-time quantitative PCR assay (sensitivity threshold: 1000 copies/mL [21] or 500 copies/mL [22]).

HIV-related markers of replication and immunostatus were collected at each study visit for all study phases. HIV-1 VLs were measured using either a branched-DNA technique (b-DNA Quantiplex 3.0, detection limit: 50 copies/mL, Bayer Diagnostics) or real-time PCR technique (COBAS AmpliPrep/COBAS TaqMan HIV-1 Test, detection limit: 40 copies/mL, Roche Molecular Systems). CD4+ T cell counts were quantified using standard measurements, while nadir CD4+ cell counts were obtained from patient records before inclusion in the first phase.

A complete hepatic battery was performed at each study visit for all phases and included quantification of the following using standard methods: alanine aminotransferase, aspartate aminotransferase, gamma-glutamyl transferase, alkaline phosphatase, total and conjugated bilirubin, alpha-fetoprotein, ferritin, albumin, haptoglobin, α2-macroglobulin, prothrombin time, and activated partial thromboplastin time. Hyaluronic acid was measured using an enzyme-linked protein-binding assay (Hyaluronic Acid Test Kit, Corgenix).

A complete cardiovascular battery was performed at each study visit for all phases and included the following markers quantified using standard methods: apolipoprotein A1, total cholesterol, high-density lipid cholesterol, low-density lipid cholesterol, and triglycerides.

#### Clinical Measures

Height, weight, and systolic and diastolic blood pressure were collected at each study visit for all phases as part of routine care. Alcohol consumption was assessed at inclusion and every 12 months in the first and second phases and at the third phase cross-sectional visit. Patients were asked whether they drank alcohol and, if so, how many glasses per day, week, or month were consumed on average over the past year.

Abdominal echography was performed at the physician’s discretion during all phases. The following information was extracted from patient files: presence of ascites; presence, number, and size of nodules; presence of portal thrombosis; presence of hepatomegaly; presence of steatosis; and evidence of dysmorphic liver.

#### Treatment

Data on antiretroviral and anti-HBV treatments, including the specific agent, start and stop dates, doses, and reason for discontinuation, were obtained during all phases. Data on all concomitant treatment, which included the same information as antiviral treatment, were also obtained during the first and second phase; however, data on only a specific set of concomitant treatments (given by Anatomical Therapeutic Chemical [ATC] Classification codes in Multimedia Appendix 1) were obtained during the third phase retrospective visits and at the third phase cross-sectional visit. All treatments were classified using the ATC codes [23]. Treatment information was retrieved from patient files and verified by the treating physician.

#### Clinical Events

Data on clinical events, including date of presentation, date of resolution (if applicable), and necessary intervention were obtained during all phases. Data on all clinical events were collected during the first and second phase; however, data on only a specific set of clinical events (given by International Classification of Diseases [ICD]-10 codes in Multimedia Appendix 2) were collected during the third phase retrospective visits and at the third phase cross-sectional visit. All clinical events were classified using the ICD-10 codes [24]. Information on clinical events was retrieved from patient files and verified by the treating physician.

### Data Management

Data were collected using paper clinical report forms, which were filled out by trained clinical research associates. Data for the third phase retrospective and third phase cross-sectional visits were manually entered in a centralized database maintained at INSERM UMRS_1136, Hôpital Saint-Antoine. To ensure data validity, we used a 10% random sample of study participants and double-entered their data. For any data field with >5% discordance between data entries, the entire data field was reverified, and discrepancies were resolved with the data manager.

### Statistical Analysis

We described the characteristics of the study population at cohort inclusion using counts and proportions or medians and IQRs. We then compared characteristics at cohort inclusion between those participating versus not participating in the third phase cross-sectional visit using a Kruskal-Wallis test for continuous variables and a Pearson *χ*^2^ test or Fisher exact test for categorical variables.

After combining vital status from study centers and external data sources, we categorized cohort participants as follows: (1) those completing the third phase cross-sectional visit, (2) discontinuation of follow-up due to death (as determined during follow-up or by linked data from CépiDC), (3) lost to follow-up with known alive vital status (as determined by clinical outpatient records obtained from the treating physician or by linked data from CépiDC), or (4) lost to follow-up with unknown vital status. We then compared participant characteristics at cohort inclusion between groups using the same statistical tests as described above.

Statistical analysis was performed using Stata software (version 15.0), and significance was determined using a *P* value <.05.

## Results

### Recruitment Flow of Participants in the Third Phase Cross-sectional Study Visit

**Figure 1:**
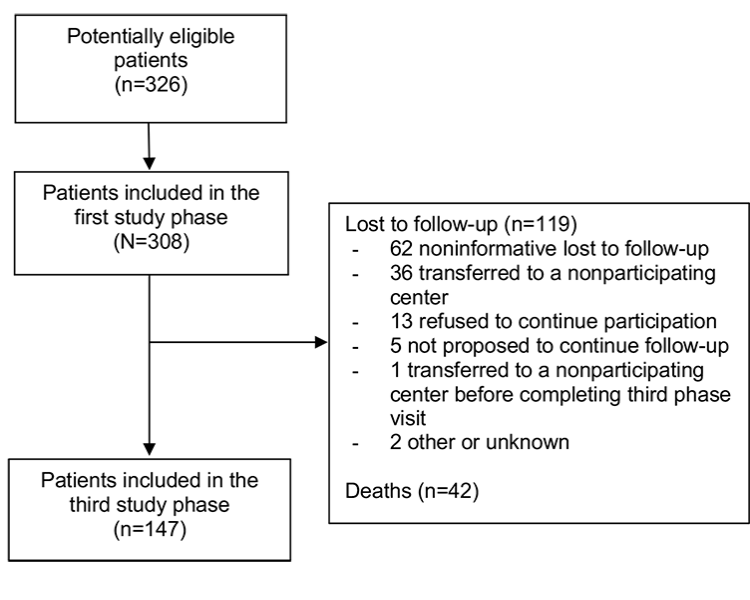
Patient flow. Patient numbers are given between the first and third phases of the French HIV–hepatitis B virus cohort. Reasons for study discontinuation between these phases are also provided. Seven individuals initially considered as lost to follow-up were in fact deceased based on date linked to the CépiDS database.

Of the 308 patients included in the first study phase, 142 (46.1%) were no longer followed at the participating center due to death (35/142, 24.6%), lost to follow-up (69/142, 48.5%), followed by a nonparticipating center (36/142, 25.3%), incarcerated (1/142, 0.7%), or unknown reasons (1/142, 0.7%). Of the 166 patients still in care at the participating center, 5 (3.5%) were not proposed to participate in the third phase cross-sectional visit and 13 (7.8%) refused participation. The remaining 148 patients provided informed consent to participate in the third phase cross-sectional study visit. One patient continued care in a nonparticipating center after signing consent and did not complete their visit, so 147 patients were considered to have completed the third phase cross-sectional visit. Patient flow from the beginning of the cohort inclusion until the third phase cross-sectional visit is summarized in Figure 1.

### Participants of the Third Phase Cross-sectional Study Visit and Their Characteristics

Of the 147 patients participating, most were male (119/147, 80.9%) with a median age of 55 years (IQR 49-59) at the time of the third phase cross-sectional visit. The median time since the first positive HIV and HBsAg tests was 24 years (IQR 18-28) and 22 years (IQR 17-26), respectively. Immunosuppression was for the most part mild with median CD4+ cell count at 548/mm^3^ (IQR 426-719), and HIV RNA was predominately undetectable (136/139, 97.8% with available data). Most patients were HBeAg negative (111/134, 82.8% with available data) and many had undetectable HBV DNA (124/132, 93.9% with available data) with a median HBV-DNA VL of 2.53 log_10_ IU/mL (IQR 2.53-2.95) for those with detectable HBV DNA. Most patients (114/147, 77.6%) underwent either TFV or TFV alafenamide–containing ART.

When comparing patients who did and did not participate in the third phase cross-sectional visit (Table 2), the only difference in inclusion characteristics observed was a lower proportion with an AIDS-defining illness (30/147, 20.4% vs 49/161, 30.4%, respectively; *P*=.04).

**Table 2:** Characteristic of the study population at cohort inclusion, stratified by participation in the third phase cross-sectional study visit.

Characteristics		Phase 1 (N=308)	Phase 3			*P* value^a^	
			Participating^b^ (n=147)	Nonparticipating (n=161)			
Male, n (%)		259 (84.1)	119 (81.0)	140 (87.0)	.15		
Age (years), median (IQR)		40 (35-45)	40 (35-44)	39 (35-45)	.93		
Alcohol consumption >1 glass/day (n=295), n (%)		176 (59.7)	87 (61.2)	89 (58.2)	.59		
**BMI (kg/m^2^; n=291), n (%)**							.94
	Underweight (16.5-18.5)	16 (5.5)	8 (5.8)	8 (5.3)			
	Normal (18.5-25.0)	221 (75.9)	105 (75.5)	116 (76.3)			
	Overweight (25-30)	46 (15.8)	23 (16.6)	23 (15.1)			
	Moderate or severe obesity (>30)	8 (2.8)	3 (2.2)	5 (3.3)			
Estimated HIV infection duration (years), median (IQR)		9.9 (3.6-14.0)	9.4 (3.8-13.1)	10.2 (3.6-14.6)	.29		
AIDS-defining illness, n (%)		79 (25.6)	30 (20.4)	49 (30.4)	.04		
CD4+ T cell count (per mm^3^), median (IQR)		400 (269-555)	404 (283-557)	399 (264-554)	.83		
Nadir CD4+ T cell count (per mm^3^; n=271), median (IQR)		212 (103-325)	212 (107-309)	215 (90-344)	.81		
ART^c^ experienced at inclusion, n (%)		251 (81.8)	122 (83.0)	129 (80.6)	.59		
Detectable HIV RNA (>50 copies/mL), n (%)		145 (47.2)	64 (43.5)	81 (50.6)	.21		
HIV-RNA viral load (log_10_ copies/mL)^d^, median (IQR)		3.90 (2.59-4.44)	3.91 (2.48-4.41)	3.89 (2.87-4.50)	.68		
From country of high endemicity, n (%)		86 (27.9)	47 (32.0)	39 (24.2)	.16		
Estimated HBV^e^ infection duration (years), median (IQR)		6.1 (2.2-10.8)	7.3 (2.8-11.2)	5.3 (2.2-10.4)	.14		
HBV DNA >60 IU/mL, n (%)		238 (77.2)	113 (76.9)	124 (77.5)	.90		
HBV DNA viral load (log_10_ IU/mL)^d^, median (IQR)		4.26 (2.55-6.58)	3.39 (2.36-6.58)	4.68 (2.66-6.69)	.12		
HBeAg^f^ positive, n (%)		160 (52.0)	73 (49.7)	87 (54.0)	.44		
**HBV genotype (** **n** **=170), n (%)**							.18
	A	105 (61.8)	51 (68.0)	54 (56.8)			
	B	1 (0.6)	0 (0.0)	1 (1.1)			
	D	17 (10.0)	7 (9.3)	10 (10.5)			
	E	19 (11.2)	8 (10.7)	11 (11.6)			
	G	19 (11.2)	5 (6.7)	14 (14.7)			
	Mixed A/D or A/G	9 (5.3)	4 (5.3)	5 (5.3)			
Precore W28stop mutation (n=164), n (%)		47 (28.7)	18 (24.7)	29 (31.9)	.31		
Lamivudine-resistance mutations (n=146), n (%)		97 (66.4)	42 (64.6)	55 (67.9)	.67		
**Other viral hepatitis, n (%)**							.48
	Anti-HCV^g^ positive serology	19 (6.2)	9 (6.1)	10 (6.2)			
	Anti-HDV^h^ positive serology	12 (3.9)	6 (4.1)	6 (3.7)			
	Anti-HCV and anti-HDV positive serology	12 (3.9)	3 (2.0)	9 (5.6)			

^a^Significance was determined using the Kruskal-Wallis test for continuous variables and Pearson *χ*² test or Fisher exact test for categorical variables.

^b^Participating patients were defined as those signing written informed consent and completing the third phase cross-sectional study visit. All characteristics reported in the table are from data collected at the inclusion visit of the French HIV-HBV cohort (2002-2003).

^c^ART: antiretroviral therapy.

^d^Among patients with detectable viral loads.

^e^HBV: hepatitis B virus.

^f^HBeAg: hepatitis B *e* virus antigen.

^g^HCV: hepatitis C virus.

^h^HDV: hepatitis D virus.

### Lost to Follow-up and Deceased Participants of the Cohort Study and Their Characteristics

Of the 161 patients not followed up at the third phase cross-sectional visit, we were able to establish vital status for the 55 patients who were already known to have been deceased (n=35), incarcerated (n=1), not proposed or refused to participate (n=5 and 13, respectively), or did not complete the third phase study visit (n=1). The remaining 106 patients not followed at the third phase cross-sectional visit did not have known vital status because of an unknown reason for lost to follow-up (n=69), being followed at a nonparticipating center (n=36), or unknown reason for noninclusion (n=1). Of these, 28 patients were able to be linked to the CépiDC database: 21 were still known to be alive and 7 were identified as deceased from the death certificate or notification registry. These deaths occurred between 2007 and 2015.

From a total of 308 participants, 147 (47.7%) participants completed the third phase cross-sectional visit, 42 (13.6%) discontinued follow-up due to death, 41 (13.3%) were lost to follow-up with known alive vital status, and 78 (25.3%) were lost to follow-up with unknown vital status. When comparing characteristics at the inclusion visit of the cohort, there were several significant differences across these patient groups (Table 3). Most differences were observed in deceased individuals who, respectively compared with all others, were significantly more likely to be older (median age 44 vs 40 years; *P*<.001), were not from a country of high HBV endemicity (4/42, 10% vs 82/266, 30.8%; *P*=.003), have a longer duration since first HIV-positive serology (median 13.1 vs 9.4 years; *P*<.001), have a higher proportion with AIDS-defining illness (21/42, 50% vs 58/266, 21.8%; *P*<.001), and have a lower nadir CD4+ cell count (median 128 vs 221 per mm^3^; *P*=.03). No significant differences in characteristics at cohort inclusion were observed between individuals completing the third phase cross-sectional visit versus lost to follow-up with known vital status. There were also no significant differences in characteristics at cohort inclusion between individuals completing the third phase cross-sectional visit versus lost to follow-up with unknown vital status, with the exception of longer duration since first HBsAg-positive serology (median 7.3 vs 3.9 years, respectively; *P*=.009) and higher proportion with detectable HIV RNA (45/78, 58% vs 64/147, 43.5%, respectively; *P*=.04).

**Table 3:** Characteristics of the study population at cohort inclusion, stratified by those completing the third phase cross-sectional visit, deceased, and lost to follow-up with known or unknown vital status.

Characteristics				Completed follow-up (n=147)		Deceased^a^ (n=42)		Lost to follow-up (n=119)					*P* value^b^	
								Known vital status^c^ (n=41)		Unknown vital status (n=78)				
Male, n (%)				119 (81.0)		39 (92.9)		35 (85.4)		66 (84.6)		.32		
Age (years), median (IQR)				40 (35-44)		44 (38-53)		39 (34-41)		38 (34-43)		.002		
Alcohol consumption >1 glass per day (n=295), n (%)				87 (61.3)		22 (55.0)		22 (55.0)		45 (61.6)		.80		
**BMI (kg/m^2^; n=291), n (%)**														.99
		Underweight (16.5-18.5)		8 (5.8)		3 (7.5)		1 (2.5)		4 (5.6)				
		Normal (18.5-25.0)		105 (75.5)		31 (77.5)		31 (77.5)		54 (75.0)				
		Overweight (25-30)		23 (16.6)		5 (12.5)		7 (17.5)		11 (15.3)				
		Moderate or severe obesity (>30)		3 (2.2)		1 (2.5)		1 (2.5)		3 (4.2)				
Estimated HIV infection duration (years), median (IQR)				9.4 (3.8-13.1)		13.1 (7.1-15.6)		10.1 (2.8-14.6)		9.4 (3.2-13.8)		.06		
AIDS-defining illness, n (%)				30 (20.4)		21 (50.0)		12 (29.3)		16 (20.5)		.001		
CD4+ T cell count (per mm^3^), median (IQR)				404 (283-557)		370 (249-474)		411 (338-619)		406 (268-562)		.65		
Nadir CD4+ T cell count (per mm^3^; n=271), median (IQR)				212 (107-309)		128 (65-304)		235 (172-346)		238 (70-384)		.08		
ART^d^ experienced at inclusion, n (%)				122 (83.0)		37 (88.1)		31 (75.6)		61 (79.2)		.45		
Detectable HIV RNA (>50 copies/mL), n (%)				64 (43.5)		14 (34.2)		22 (53.7)		45 (57.7)		.05		
HIV-RNA viral load (log_10_ copies/mL)^e^, median (IQR)				3.91 (2.48-4.41)		3.83 (2.59-4.05)		3.99 (3.04-4.53)		3.67 (2.87-4.46)		.89		
From country of high endemicity, n (%)				47 (32.0)		4 (9.5)		13 (31.7)		22 (28.2)		.02		
Estimated HBV^f^ infection duration (years), median (IQR)				7.3 (2.8-11.2)		6.7 (2.3-13.5)		5.5 (2.8-11.5)		3.9 (1.7-8.9)		.04		
HBV DNA >60 IU/mL, n (%)				113 (76.9)		31 (75.6)		31 (75.6)		62 (79.5)		.95		
HBV-DNA viral load (log_10_ IU/mL)^e^, median (IQR)				3.39 (2.36-6.58)		4.63 (2.71-6.88)		5.02 (2.28-6.58)		4.54 (2.76-6.88)		.45		
HBeAg^g^ positive, n (%)				73 (49.7)		29 (69.0)		20 (48.8)		38 (48.7)		.13		
**HBV genotype (n=170), n (%)**														.36
		A		51 (68.0)		17 (60.7)		13 (61.9)		24 (52.2)				
		B		0 (0.0)		0 (0.0)		0 (0.0)		1 (2.2)				
		D		7 (9.3)		4 (14.3)		2 (9.5)		4 (8.7)				
		E		8 (10.7)		1 (3.6)		1 (4.8)		9 (19.6)				
		G		5 (6.7)		4 (14.3)		4 (19.1)		6 (13.0)				
		Mixed A/D or A/G		4 (5.3)		2 (7.1)		1 (4.8)		2 (4.3)				
Precore W28stop mutation (n=164), n (%)				18 (24.7)		4 (15.4)		7 (33.3)		18 (40.9)		.10		
Lamivudine-resistance mutations (n=146), n (%)				42 (64.6)		14 (66.7)		14 (77.8)		27 (64.3)		.80		
**Other viral hepatitis, n (%)**														.31
	Anti-HCV^h^ positive serology		9 (6.1)		2 (4.8)		4 (9.8)		4 (5.1)					
		Anti-HDV^i^ positive serology		6 (4.1)		1 (2.4)		3 (7.3)		2 (2.6)				
		Anti-HCV and anti-HDV positive serology		3 (2.0)		5 (11.9)		1 (2.4)		3 (3.9)				

^a^Includes deaths observed during the study and from vital records.

^b^Overall significance between groups determined using the Kruskal-Wallis test for continuous variables and Pearson *χ*² test or Fisher exact test for categorical variables.

^c^Includes patients whose vital status is known: 13 refusing to continue participation, 5 not proposed to continue follow-up, 1 transferred to a nonparticipating center before completing the third phase visit, 2 other or unknown reasons, and 22 with vital status determined by CépiDC.

^d^ART: antiretroviral therapy.

^e^Among patients with detectable viral loads.

^f^HBV: hepatitis B virus.

^g^HBeAg: hepatitis B *e* virus antigen.

^h^HCV: hepatitis C virus.

^i^HDV: hepatitis D virus.

## Discussion

### Principal Findings

A handful of prospective cohort studies have examined the effect of HIV-HBV coinfection on mortality outcomes [10], but most of these studies have incomplete data on HBV status, making it difficult to determine how HBV infection contributes to HBV-related disease. We present the design of a recent extension of a large observational cohort of patients coinfected with HIV-HBV in France to address the shortcomings of these previous cohorts.

### Strengths and Weaknesses

One of the major strengths of our cohort is the consistent collection of HBV-DNA VLs, complete HBV serological battery, HBV genetic characterization (specifically regarding antiviral resistance mutations in the *pol* gene, vaccine escape mutations in the *S* gene, and W28* *precore* mutations [25]), and liver fibrosis measurements. To our knowledge, few cohorts have regularly collected these data and those that have this information are no longer in active follow-up [26,27]. The regular collection of markers of HBV replication and HBV serological data will help provide more precise estimates of when certain events occur (eg, time to undetectable HBV-DNA or HBeAg or HBsAg seroclearance) as well as their durability (eg, persistent HBV-DNA viremia or sustained HBeAg or HBsAg seroclearance) or variability (as was observed for noninvasive measures of liver fibrosis [28]). At the same time, we aim to associate these primary outcomes with all-cause mortality.

In addition, by extending our previous cohort, we collected data for up to 15 years of follow-up. There are certain facets of HIV-HBV coinfection that make data sets containing longer follow-up important. HBsAg seroclearance is a slow process [29] and is achieved in approximately 1% per year in treated coinfected patients [17]. TFV-containing ART regimens have shown effective antiviral potency [4]. However, long-term toxicity issues related to treatment and/or comorbidities among specifically coinfected patients with HIV-HBV may hamper its clinical effectiveness. It is also unknown how HIV-HBV coinfection evolves in an aging population, especially with respect to severe non-AIDS and non-HBV-related clinical events. The longer follow-up provided in the French HIV-HBV cohort could help establish the frequency of these end points and their determinants, provided that there are a sufficient number of events.

Another advantage of our cohort is the availability of frozen samples dating back to the beginning of the cohort. As these samples have been stored at a centralized location, they have facilitated their use in several collaborations, some of which have involved the genetic variability of HBV [25,30], compared with patients undergoing intensification with pegylated-interferon [31], HDV-RNA replication in tri-infected patients [32], kinetics of HBsAg or HBeAg quantification [33], hepatitis B core-related antigen quantification [34], and covalently closed circular DNA [35] during TFV treatment and ongoing projects related to markers of replication during treatment. Nevertheless, it should be mentioned that stored samples are only available in the first and second phases and at the third phase cross-sectional visit. As a result, any future analysis using available samples will have a gap in follow-up time.

There are other limitations in the French HIV-HBV cohort. First, most patients had years of ART experience before inclusion in the first phase, were HBeAg positive, came from predominately Western Europe, and sexually acquired HBV. The vast majority of individuals with HIV currently diagnosed with an HBV coinfection in Europe come from regions of high HBV endemicity, while new HBV infections have declined dramatically in other Western European settings [36,37]. Therefore, our study population may not be fully representative of modern coinfected patients. Second, despite the large number of patients and extensive follow-up, we might not have enough end points to ensure sufficiently powered comparisons. Third, certain data were not consistently collected during follow-up (eg, TE measurements only began at the start of the second phase) or across the entire study population (eg, HBsAg levels are only available in the first 2 phases among TFV-treated patients). Fourth, metabolic data (insulin, adiponectin, controlled attenuation parameter of the FibroScan, etc) were not collected at any point during follow-up, making it difficult to understand the increasing role of metabolic disorders in this patient population [38].

### Assessment of Participation in the Third Phase Study Visit and Loss to Follow-up

Similar to other longitudinal studies, one major concern is loss to follow-up and the possible biases induced thereto. At the third phase of follow-up, occurring roughly 15 years after the start of the cohort, almost half of the patients did not continue follow-up. When compared with rates of loss to follow-up in HIV-positive (5.4% of patients lost to follow-up from 1999 to 2009 in the French Hospital Database on HIV study [39]) or HIV-HCV coinfected (10% of patients lost to follow-up from 2006 to 2010 in the Agence Nationale de Recherche sur le Sida et les Hépatites (ANRS) CO13 HEPAVIH study [40]) cohorts from France, loss to follow-up would seem to occur at a much higher rate in our cohort.

Individuals lost to follow-up could have a higher risk of more advanced disease (opportunistic infections, advanced liver fibrosis or cirrhosis, cancer, advanced cardiovascular diseases, etc), which might make them less willing to participate or continue follow-up. No particular population characteristic at cohort inclusion was significantly different between participating and nonparticipating patients across phases, except for a higher proportion of AIDS-defining illnesses in those not participating in the third phase cross-sectional visit. There could also be other characteristics during follow-up, either measured or unmeasured (eg, nonreported comorbidities) in our cohort that were associated with loss to follow-up. This might induce differential loss to follow-up bias, the effect of which depends on the research question addressed. Therefore, the assessment of this bias is imperative in future studies from our cohort.

Previous studies linking data from the French Hospital Database on HIV study and other mortality outcome registries or cohorts demonstrated that 29.8% of patients with HIV who were lost to follow-up had in fact died [41]. In our cohort, this proportion was similar at 32% (7/22), and notwithstanding the fact that 61.9% (78/126) of individuals with lost to follow-up were unable to have their vital status obtained is comparable with the proportion deceased of those remaining in follow-up (35/182, 19.2% either died or participated in the third phase cross-sectional visit).

### Conclusions

Long-term and more comprehensive cohorts of coinfected individuals with HIV-HBV are pivotal in understanding how liver-related and nonliver-related morbidity and mortality can develop in this patient population. We were able to extend our cohort data on HBV replication, HBV serology, and liver fibrosis to up to 15 years; however, given the fact that approximately half of the patients were unable to participate in the third phase cross-sectional study, either due to death or lost to follow-up, the biases associated with this high rate of loss to follow-up need to be considered in future studies from this cohort. Nevertheless, these data will be helpful in enhancing our knowledge of the clinical trajectory during coinfection. More specifically, the clinical implications of persistent viremia, lack of HBeAg or HBsAg seroclearance, and progressive development of liver fibrosis will be evaluated along with their association with all-cause mortality. The French HIV-HBV cohort may also contribute to future collaborations aimed at assessing rarer outcomes, such as hepatic decompensation, hepatocellular carcinoma, and cause-specific death.

## Appendix

Multimedia Appendix 1 Classes of concomitant treatment collected during the third phase cross-sectional study visit.

Multimedia Appendix 2 Types of clinical events collected during the third phase cross-sectional study visit.

## Abbreviations

ANRS: Agence Nationale de Recherche sur le Sida et les Hépatites
ART: antiretroviral therapy
ATC: Anatomical Therapeutic Chemical
HBeAg: hepatitis B *e* antigen
HBsAb: hepatitis B surface antibody
HBsAg: hepatitis B surface antigen
HBV: hepatitis B virus
HCV: hepatitis C virus
HDV: hepatitis D virus
ICD: International Classification of Diseases
IU: international unit
PCR: polymerase chain reaction
TFV: tenofovir
TE: transient elastography
VL: viral load
