# Supplementary material for: Subclinical and Clinical Outcomes in Patients Coinfected With HIV and Chronic Hepatitis B Virus From Clinical Outpatient Centers in France: Protocol for an Ambispective, Longitudinal Cohort Study
Source: JMIR Res Protoc. 2021 Apr 6;10(4):e24731. doi: 10.2196/24731 (PMC8058690; doi:10.2196/24731)
Supplement: Multimedia Appendix 1 [file resprot_v10i4e24731_app1.docx]

**Supplementary Table 1. Classes of concomitant treatment collected during the third phase**

| **Anatomical main group** | **ATC code*** | **Pharmacological subgroup** |
| --- | --- | --- |
| Alimentary tract and metabolism | A05A | bile therapy |
|  | A05B | liver therapy, lipotropics |
|  | A10A | insulins and analogues |
|  | A10B | blood glucose lowering drugs, excluding insulins |
|  | A10X | Other drugs used in diabetes |
| Blood and blood forming organs | B01A | antithrombotic agents |
|  | B02A | antifibrinolytics |
|  | B02B | vitamin K and other hemostatics |
| Cardiovascular system | C01A | cardiac glycosides |
|  | C01B | antiarrhythmics, class I and III |
|  | C01C | cardiac stimulants excluding cardiac glycosides |
|  | C01D | vasodilators used in cardiac diseases |
|  | C01E | other cardiac preparations |
|  | C02 | antihypertensives |
|  | C03 | diuretics |
|  | C04A | peripheral vasodilators |
|  | C07A | beta-blocking agents |
|  | C08 | calcium channel blockers |
|  | C09 | agents acting on the renin-angiotensin system |
|  | C10 | lipid modifying agents |
| Antineoplastic and immunomodulating agents | L01A | alkylating agents |
|  | L01B | antimetabolites |
|  | L01C | plant alkaloids and other natural products |
|  | L01X | other antineoplastic agents |
|  | L03A | immunostimulants |
|  | L04A | immunosuppressants |

*Data were collected on any agents whose ATC code began with the letters/numbers presented in this table.
