# Supplementary material for: Subclinical and Clinical Outcomes in Patients Coinfected With HIV and Chronic Hepatitis B Virus From Clinical Outpatient Centers in France: Protocol for an Ambispective, Longitudinal Cohort Study
Source: JMIR Res Protoc. 2021 Apr 6;10(4):e24731. doi: 10.2196/24731 (PMC8058690; doi:10.2196/24731)
Supplement: Multimedia Appendix 2 [file resprot_v10i4e24731_app2.docx]

**Supplementary Table 2. Types of clinical events collected during the third phase**

| **Broad category** | **ICD-10 code*** | **Blocks of three-character categories** |
| --- | --- | --- |
| Certain infectious and parasitic diseases | A00-A09 | Intestinal infectious diseases |
|  | A20-A28 | Certain zoonotic bacterial diseases |
|  | A30-A49 | Other bacterial diseases |
|  | A50-A64 | Infections with a predominantly sexual mode of transmission |
|  | A65-A69 | Other spirochetal diseases |
|  | A70-A74 | Other diseases caused by chlamydia |
|  | A75-A79 | Rickettsioses |
| Neoplasms | C00-C75 | Malignant neoplasms, stated or presumed to be primary, of specified sites, except of lymphoid, hematopoietic and related tissue |
|  | C81-C96 | Malignant neoplasms, stated or presumed to be primary, of lymphoid, hematopoietic and related tissue |
| Endocrine, nutritional and metabolic diseases | E10-E14 | Diabetes mellitus |
|  | E15-E16 | Other disorders of glucose regulation and pancreatic internal secretion |
| Diseases of the circulatory system | I00-I02 | Acute rheumatic fever |
|  | I05-I09 | Chronic rheumatic heart diseases |
|  | I10-I15 | Hypertensive diseases |
|  | I20-I25 | Ischemic heart diseases |
|  | I60-I69 | Cerebrovascular diseases |
|  | I70-I79 | Diseases of arteries, arterioles and capillaries |
| Diseases of the digestive system | K70-K77 | Diseases of liver |
|  | K80-K87 | Disorders of gallbladder, biliary tract and pancreas |
| Diseases of the musculoskeletal system and connective tissue | M80-M85 | Disorders of bone density and structure |
|  | M86-M90 | Other osteopathologies |
| Diseases of the genitourinary system | N00-N08 | Glomerular diseases |
|  | N10-N16 | Renal tubulo-interstitial diseases |
|  | N17-N19 | Renal failure |
|  | N25-N29 | Other disorders of kidney and ureter |

*Data were collected on any event whose ICD-10 code began with the numbers/letters presented in this table.
